# Growths in the Nasal Passages Due to Colds

**Published:** 1896-10

**Authors:** 


### Growths in the Nasal Passages Due to Colds.

Dr. Thomas F. Rumbold says every patient in whom growths are found has a history of taking colds in the head for years before he noticed the nasal impediment. These growths must have a cause, and the only ailment the patient had was his nasal colds—hereditary of disease cuts no figure—therefore, the inflammation caused by the colds is the cause of the growths. Long and careful observation will compel every one to admit this. I am well aware that not infrequently a patient who has a large growth will say: "I do not take cold easily or frequently. I do not think I have had a cold in the head for two or three years until the one taken a few weeks ago." The reason for this apparent absence of colds is that his mucous membrane has been so long under the influence of inflammation—certainly several years before his nasal growths appeared—that the membrane is in an anæsthetic condition; obtunded sensibility of a part always follows a chronic inflammation, so that the patient cannot feel the effect of slight colds; it requires the effect of a severe cold to cause his mucous membrane to recognize it. Nor does this disagreeable effect last very long, so that he is quite liable to forget the last cold. It will be noticed that the older such patients, the less they will say about taking colds in the head, only because their mucous membrane is becoming more and more obtunded; but it will be noticed, nevertheless, that they are now far more cautious to avoid a draught of air, and they wear much warmer clothing than they did several years previous, showing plainly that the whole system, unknown to them by any sensation they experience, is becoming more and more under cold-taking influence.—*St. Louis Med. and Surg. Journal.*

---

The British Medical Association has 16,332 members. The entire number of medical practitioners in the Kingdom is 27,392.

---

Cold burns like molten metal at a temperature of 180 degrees below zero.

